# Neoadjuvant immunotherapy and chemoimmunotherapy for stage II-III muscle invasive bladder cancer

**DOI:** 10.3389/fimmu.2022.986359

**Published:** 2022-08-17

**Authors:** Hualin Chen, Wenjie Yang, Xiaoqiang Xue, Yingjie Li, Zhaoheng Jin, Zhigang Ji

**Affiliations:** Department of Urology, Peking Union Medical College Hospital, Chinese Academy of Medical Science and Peking Union Medical College, Beijing, China

**Keywords:** neoadjuvant, immunotherapy, chemoimmunotherapy, muscle-invasive bladder cancer (MIBC), meta-analysis

## Abstract

**Objective:**

Considering the striking evidence revealed by immunotherapy in advanced or metastatic bladder cancer, investigators have explored neoadjuvant immunotherapy and chemoimmunotherapy in muscle-invasive bladder cancer (MIBC). Currently, there have been a large number of studies reporting varied efficacy and safety of these approaches. Herein, we pooled the available evidence in terms of oncological outcomes (pathological complete response [pCR] and pathological partial response [pPR]) and safety outcomes (immune-related adverse events [irAEs], treatment-related adverse events [TRAEs]), through a systematic review and meta-analysis.

**Method:**

We searched PubMed, Embase, Cochrane Library, and American Society of Clinical Oncology meeting abstracts to identify relevant studies up to June 2022. Studies were included if they evaluated the neoadjuvant immunotherapy or chemoimmunotherapy in MIBC and reported at least the pCR.

**Results:**

A total of 22 records involving 843 patients were included. For pCR of immunotherapy, the pooled rate of immune checkpoint inhibitor (ICI) monotherapy and dual-ICIs therapy was 24% (95% confidence interval [CI]: 15.3% - 32.8%) and 32.1% (95%CI: 20.6% - 43.7%), respectively. For pCR of chemoimmunotherapy, the overall pooled rate was 42.6% (95% CI: 34.9% - 50.2%). Subgroup of gemcitabine/cisplatin (GC) plus ICI had a pCR rate of 41.7% (95%CI: 35.8% - 47.5%). In terms of safety, the pooled rate of Grade≥3 irAEs was 11.7% (95% CI: 6.5%-16.9%). In subgroup analysis, the Grade≥3 irAEs rate of ICI monotherapy, dual-ICIs therapy, and GC plus ICI therapy was 7.4% (95% CI: 4.3%-10.5%), 30.3% (95% CI: 15.3%-45.3%), and 14.5% (95% CI: 3.5% - 25.4%), respectively. Besides, the pooled Grade≥3 TRAEs rate for chemoimmunotherapy was 32.4% (95% CI: 13.1% - 51.6%).

**Conclusion:**

Neoadjuvant immunotherapy and chemoimmunotherapy were effective and safe in the treatment of MIBC. Compared to ICI monotherapy, dual-ICIs therapy or chemoimmunotherapy can improve the response rate, while increasing the morbidity of Grade≥ 3 irAEs or Grade≥ 3 TRAEs.

**Systematic Review Registration:**

https://www.crd.york.ac.uk/prospero/, identifier CRD4202233771.

## Introduction

Bladder cancer is one of the most common urological malignancies worldwide, with newly diagnosed around 500 000 cases and 200 000 deaths each year (1). Based on the involvement of the bladder muscle or not, bladder cancer can be generally classified into muscle-invasive (MIBC) and non-muscle-invasive bladder cancer (NMIBC), each with distinct biological features and prognosis. NMIBC accounts for approximately 70% of newly diagnosed cases and denotes a favorable prognosis, with 5-year overall survival (OS) rate approaching 90% (2). The remaining 20% and 10% of cases present with muscle-invasive features and advanced stage, and suffer from poor prognosis, with the 5-year OS rate decreasing to 60% and less than 30%, respectively (3). Besides, the dissemination features dramatically increase the metastatic risk, resulting in a 5-year OS rate plunging to 6% (2). Therefore, effective management of MIBC at an early stage can attenuate the risk of local or distant spread and benefit survival.

Neoadjuvant chemotherapy (NAC) followed by radical cystectomy (RC) represents the standard of care (SoC) for cisplatin-eligible MIBC, remarkably improving the OS. To be specific, for MIBC patients achieving a pathological partial response (pPR) at the time of RC, the 5-year OS rate can approach 90% (2). However, this attractive therapeutic strategy fails to meet the needs of cisplatin-ineligible MIBC, as defined by a previous study (4). Furthermore, a partial of cisplatin-eligible MIBC patients may suffer from severe treatment-related adverse effects and have to discontinue the treatment protocol.

Thanks to the in-depth knowledge of molecular mechanisms of immune checkpoints in the resistance to anti-tumor immunity, immune checkpoint inhibitors (ICIs) have reshaped the treatment paradigm and revolutionized the prognosis of several cancers including melanoma, renal cell carcinoma, and non–small cell lung cancer. A growing number of studies have explored the feasibility and safety of neoadjuvant ICI therapy in MIBC, considering their efficacy in the advanced stage or metastatic setting (5). In the PURE-01 study, preoperative three cycles of pembrolizumab contributed to a 37% pathological complete response (pCR) rate and 55% pPR rate (5). Inspired by these preliminary findings, investigators further explored the feasibility and safety of dual-ICIs and chemoimmunotherapy strategies, in an attempt to amplify the efficacy and promote the prognosis. Van Dijk et al. reported a 45.8% pCR rate in the NABUCCO cohort I study in which three cycles of ipilimumab plus nivolumab were given to stage III MIBC (6). The pCR rate climbed to 51.3% in the BLASST-1 study investigating gemcitabine/cisplatin (GC) plus nivolumab (7).

Herein, we conducted a systemic review on the recent progress of neoadjuvant immunotherapy and chemoimmunotherapy in stage II-III MIBC. Although data on the role of immunotherapy plus targeted therapy for MIBC is limited, a review of current preliminary results has been performed.

## Methods and materials

We performed the study according to the Preferred Reported Items for Systematic Reviews and Meta-Analyses (PRISMA) guidelines (8). PROSPERO registration number: CRD42022337714.

### Literature research

**Figure 1:**
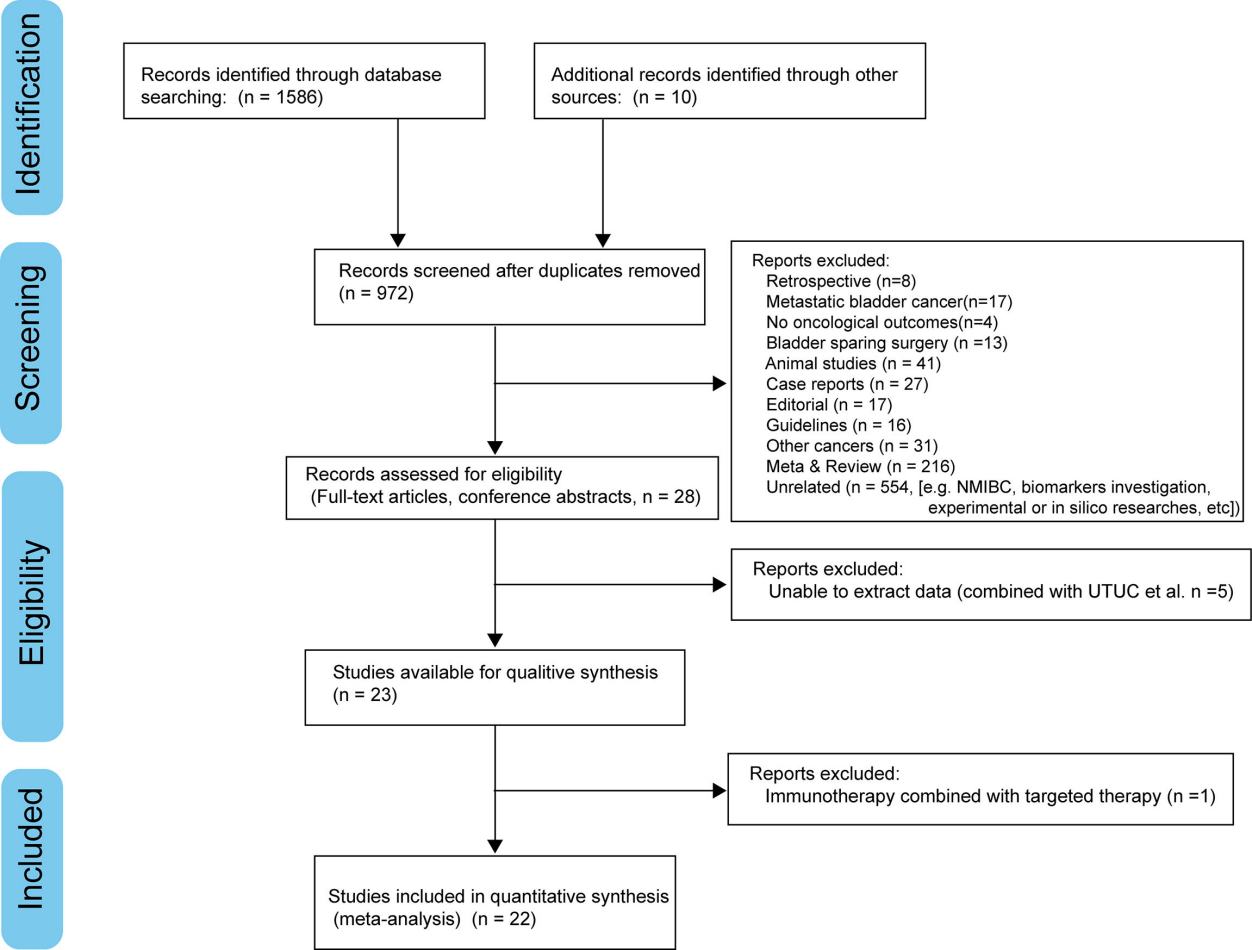
PRISMA flowchart.

We searched PubMed, Embase, Cochrane Library, and American Society of Clinical Oncology (ASCO) meeting abstracts to identify relevant studies up to June 2022. **Figure 1** demonstrates the workflow of literature identification.

The search algorithm was formulated using the following Boolean strategy: (“immunotherapy” OR “nivolumab” OR “ipilimumab” OR “atezolizumab” OR “pembrolizumab” OR “durvalumab” OR “avelumab” OR “tremelimumab” OR “immune checkpoint inhibitor” OR “chemotherapy” OR “gemcitabine” OR “cisplatin”) AND (“neoadjuvant” OR “preoperative”) AND (“urothelial carcinoma” OR “bladder cancer” OR “bladder carcinoma”).

Two authors (H.L.C. and W.J.Y.) independently reviewed the search results and any discrepancy was resolved by consulting with a third author (Z.G.J).

### Inclusion and exclusion criteria

The inclusion criteria were listed as follows:

(1) Patients with MIBC (stage II/III);
(2) Patients received at least one type of anti–PD-(L)1 or anti-CTLA4 in the neoadjuvant setting, combined with chemotherapy or not;
(3) Patients underwent RC;
(4) Oncological endpoints were reported: pCR and pPR. The pCR was defined as pathological staging = ypT0N0M0 and the pPR was defined as pathological staging ≤ ypT1N0M0 including ypT0-1N0M0 and ypTisN0M0, consistent with previous studies (9).

(1) Eligible study type: single-arm, RCTs, and non-randomized controlled studies (non-RCTs).

The exclusion criteria were listed as follows:

(1) Patients with other cancers or metastatic bladder cancer;
(2) Patients underwent bladder sparing surgery;
(3) No oncological outcomes were reported;
(4) Non-eligible study type: retrospective, animal, review, meta-analysis, case reports, editorials, guidelines.

### Data extraction

Two authors (H.L.C. and W.J.Y.) independently extracted the data and any discrepancy was resolved by consulting with a third author (Z.G.J).

Three types of data were extracted:

(5) Patients: age, cisplatin eligibility, performance status (PS) score, tumor stage, number at recruitment, and RC;
(6) Therapeutic strategy: type of immunotherapy/chemoimmunotherapy, regimen, surgical timeframe (from the last dose to RC), non-response rate (PD and stable disease [SD]), and follow-up period;
(7) Oncological and survival outcomes: pCR, pPR, recurrence-free survival (RFS), and OS;
(8) Safety outcomes: immune-related adverse events (irAEs), treatment-related adverse events (TRAEs), surgical complications, steroid requirement, and death;
(9) Study: first author, trail name/ID, trial phase, study duration, and study design.

We also contacted the corresponding authors *via* e-mail if the above-mentioned data was not available.

### Quality assessment

The modified Jadad scale was used for RCTs (10) and the methodological index for non-randomized studies (MINORS) was used for non-RCTs or single-arm studies (11).

### Statistical analysis

All included studies were prospective trials. Considering unpredictable withdrawal from the original well-designed protocol, not all enrolled patients underwent RC after neoadjuvant therapies. Therefore, we performed per protocol (PP) analysis in the pooled oncological outcomes. R version 4.2.0 (The R Foundation for Statistical Computing, MO, USA) and the “*meta*” package were utilized in the analysis (12). Owing to significant heterogeneity across these studies, we utilized the random effects model to pool these results. Publication bias was assessed by Egger’s regression test (13). Influence analysis was performed to evaluate the impact of each study on the overall pooled result.

## Results

### Records screening results and characteristics

After screening, 22 records (24 cohorts) involving 843 patients, were included in this analysis. Most studies were phase II single-arm trials and carried out within the last six years. Two RCTs scored 6 points by the modified Jadad scale and were regarded to be high-quality (14, 15). The other single-arm designed studies were assessed as acceptable for the meta-analysis, with scores ranging from 12 to 15 points by the MINORS index. The clinical tumor stage was assessed as stage II-III. 13 records involving 542 patients reported the proportion of TNM stage in detail: 65.7% of cT2, 33.4% of cT3-4a, and 2.0% of cN1. Neoadjuvant ICI monotherapy was explored in eight cohorts (two pembrolizumab, two atezolizumab, two nivolumab, and one durvalumab, and one avelumab), dual-ICIs therapy in five cohorts (three ipilimumab plus nivolumab and two durvalumab plus tremelimumab) and chemoimmunotherapy in 11 cohorts (eight gemcitabine/cisplatin [GC] plus ICI, one dose-dense course of methotrexate, vinblastine, doxorubicin, and cisplatin [ddMVAC] plus ICIs, one gemcitabine plus ICI, and one paclitaxel/gemcitabine [PG] plus ICI). **Table 1** demonstrated the characteristics in detail.

**Table 1:** Characteristics of included studies and patients.

Author	Trail ID/name	Study period	Study design	cTNM stage,cisplatin eligibility	Study arm(s)	No. of pts	Regimen, cycles	Age (median, yrs)	Gender (male, %)	Surgery timeframe	F/u	Quality
Powles (16)	NCT02662309 ABACUS	May 2016- June 2018	single-arm, phase II	T2–T4aN0M0, Cis-ineligible or refusal	Atezolizumab	95	2	73	85%		13.1	15
Koshkin (17)	NCT02451423	by October 2020	single-arm, phase II	T2-4aN0-1M0, Cis-ineligible or refusal	Atezolizumab	20	1-3	69	75%		21.4	14
Wei (18)	NCT03773666 BLASST-2	February 2019- September 2019	single-arm	T2-4aN0M0, Cis-ineligible or refusal	Durvalumab	10	3	67	80%	2-4 wks		14
Goubet (19)	NCT03212651 PANDORE	October 2017- December 2019		T2-4aN0M0	Pembrolizumab	39	3					12
Necchi (20)	NCT02736266 PURE-01	February 2017- June 2019	single-arm, phase II	T2-4aN0M0, Regardless of cis-eligibility	Pembrolizumab	114	3	66	86.8%	median 3 wks	13.2	15
Guercio (21)	NCT03520491	August 2018- May 2021	non-RCT, phase II	T2-4aN0M0, Cis-ineligible	armA: Nivolumab armB: Nivolumab + Ipilimumab	armA: 15 armB: 15		76	80%	within 60 days		13
Yin (22)	NCT03532451 PrE0807		non-RCT, phase Ib	T2-4aN0-1M0, Cis-ineligible or refusal	armA: Nivolumab armB: Nivolumab + lirilumab	armA: 13 armB: 30		75	67%	median 27days		14
Van Dijk (6)	NCT03387761 NABUCCO cohort I	February 2018- February 2019	single-arm, phase Ib/II	T2-T4aN0-1M0, Regardless of cis-eligibility	Nivolumab + Ipilimumab	24	3	65	75%		8.3	15
Van Dorp (23)	NCT03387761 NABUCCO cohort II			stage III, Cis-ineligible or refusal	Nivolumab + Ipilimumab	30	3					13
Kim (24)	KCT0003804 CRIS	September 2019- October 2020	single-arm, phase II	T2-4aN0M0, Cis-eligible	GC+ Nivolumab	51	3-4				19	14
Gupta (7)	NCT03294304 BLASST-1	February 2018- June 2019	single-arm, phase II	T2-4aN0-1M0, Cis-eligible	GC+ Nivolumab	41	4			within 8 wks	15.8	14
Funt (25)	NCT02989584	February 2018- May 2020	single-arm, phase II	T2-4aN0M0	GC+ Atezolizumab	44	4			median 7.8 wks	16.5	12
Xing (26)	ChiCTR2000032359	By April 2021	single-arm	T2-4aN0-1M0, Cis-eligible	GC+ Camrelizumab	19	3	69	73.7%	median 4.3 wks		12
Rose (27)	NCT02690558	June 2016- March 2020	single-arm, phase II	T2-4aN0-1M0	GC+ Pembrolizumab	39	4					14
Grande (14)	NCT03472274 DUTRENEO	October 2018- December 2019	RCT phase II	cT2‐4aN0-1M0, Cis-eligible	armA: Durvalumab+ Tremelimumab armB: GC/ddMVAC	armA: 23 armB: 38	3					6
Gao (28)	NCT02812420	April 2017- December 2018		T2-4aN0M0, Cis-ineligible	Durvalumab+ Tremelimumab	28	2	71	71%	within 4–6 wks	19.2	15
Kaimakliotis (29)	NCT02365766		single-arm, phase 1b/II	T2-4aN0M0, Cis-eligible	GC+ Pembrolizumab	40	4	65	75%	median 5.3 wks	17.4	14
Cathomas (30)	SAKK 06/17	July 2018- September 2019	single-arm, phase II	T2-4aN0-1M0, Cis-eligible	GC+ Durvalumab	61	4	67.5	79%		28.1	14
Thibault (31)	NCT03549715 NEMIO	December 2018- July 2019	single-arm, phase I/II		ddMVAC+ Durvalumab ± Tremelimumab	12	2	59.5		4‐8 wks		12
Hristos (32)	NCT02365766 GU14-188 cohort2			T2-4aN0M0, Cis-ineligible	Gemcitabine+ Pembrolizumab	37	3	72	70%	median 5.6 wks	10.8	13
Chanza (15)	NCT03674424 Oncodistinct 004 – AURA		RCT phase II	T2-4aN0-1M0, armA: Cis-eligible	armA: PG+ Avelumab armB: Avelumab	armA: 28 armB: 28	4	armA: 72 armB: 75	armA: 93% armB: 93%			6
Lin (33)	ChiCTR2000037670	By Oct 2021	single-arm phase II	T2-4aN0M0, Cis-eligible	GC+ Tislelizumab	17	4	62		within 6 wks		12

Cis-ineligible/Cis-eligible, cisplatin-ineligible/cisplatin-eligible; F/u, follow up. Any box left blank is related to information that has not been reported.

### Oncological outcomes

**Table 2** presented the oncological outcomes in detail.

**Table 2:** Oncological and safety outcomes. .

Author	Study arm(s)	RC pts	Oncological outcomes		Non-responder PD/SD	Safety			
			pCR, n (%)	pPR		≥ Grade 3 irAEs	≥ Grade 3 surgical complications	Steroid requirement	Tx-related death
Powles (16)	Atezolizumab	87	27 (31.0%)			10	16		1
Koshkin (17)	Atezolizumab	20	2 (10.0%)	5		2			
Wei (18)	Durvalumab	8	1 (12.5%)	2		1			
Goubet (19)	Pembrolizumab	34	10 (29.4%)						
Necchi (20)	Pembrolizumab	112	42 (37.5%)	63	1 PD 7 SD	8	25	4	
Guercio (21)	armA: Nivolumab armB: Nivolumab + Ipilimumab	armA: 11 armB: 9	armA: 2 (18.2%) armB: 1 (11.1%)	4 3	armA: 2 PD armB: 3 PD	1 4			
Yin (22)	armA: Nivolumab armB: Nivolumab + lirilumab	armA: 12 armB:29	armA: 1 (8.3%) armB: 5 (17.2%)	armA: 2 armB: 8	armA: 1 PD	armA: 0 armB: 4			
Van Dijk (6)	Nivolumab + Ipilimumab	24	11 (45.8%)	14		13			
Van Dorp (23)	Nivolumab + Ipilimumab	26	7 (26.9%)	11	1 PD				
Kim (24)	GC+ Nivolumab	34	12 (35.3%)	22					
Gupta (7)	GC+ Nivolumab	39	20 (51.3%)	27		3		0	
Funt (25)	GC+ Atezolizumab	39	16 (41.0%)	27	2 PD	5		2	
Xing (26)	GC+ Camrelizumab	11	6 (54.5%)	7	1 PD	0			
Rose (27)	GC+ Pembrolizumab	38	14 (36.8%)	22					
Grande (14)	armA: Durvalumab+ Tremelimumab armB: GC/ddMVAC	armA: 20 armB: 35	armA: 8 (40.0%) armB: 19 (54.3%)		armA: 1 PD armB: 2 PD	armA: 5 armB: 18			
Gao (28)	Durvalumab+ Tremelimumab	24	9 (37.5%)	14	5 PD, 2 SD	6		4	
Kaimakliotis (29)	GC+ Pembrolizumab	36	16 (44.4%)	22					
Cathomas (30)	GC+ Durvalumab	53	18 (34.0%)	32	1 PD	16			
Thibault (31)	ddMVAC+ Durvalumab ± Tremelimumab	12	8 (66.7%)	9		0			
Hristos (32)	Gemcitabine+ Pembrolizumab	34	18 (52.9%)	19	3 PD	4			
Chanza (15)	armA: PG+ Avelumab armB: Avelumab	armA: 27 armB: 28	armA: 5 (18.5%) armB: 10 (35.7%)	6 11	armA: 1 PD	armA: 2			
Lin (33)	CG+ Tislelizumab	17	10 (58.8%)	13					

RC, radical cystectomy; pts, patients; pCR, pathological complete response; pPR, pathological partial response; PD, progressive disease; SD, stable disease; irAEs, immune related adverse events; Tx-related, treatment-related; GC, gemcitabine/cisplatin; ddMVAC, dose-dense course of methotrexate, vinblastine, doxorubicin, and cisplatin; PG, paclitaxel/gemcitabine. Any box left blank is related to information that has not been reported.

#### pCR

**Figure 2:**
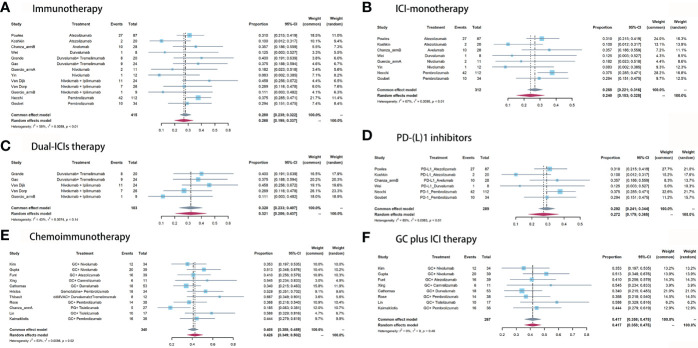
Pooled pCR of **(A)** immunotherapy, **(B)** ICI monotherapy, **(C)** dual-ICIs therapy, **(D)** PD-(L)1 inhibitors, **(E)** chemoimmunotherapy, and **(F)** GC plus ICI therapy.

For neoadjuvant immunotherapy, 12 records (eight ICI monotherapy and five dual-ICIs therapy cohorts) involving 415/454 (91.4%) RC patients were included in this analysis. Within the 12 studies, there were four PD-L1 cohorts (16–18, 34), two PD-1 cohorts (19, 20), two CTLA-4 cohorts (21, 22), three CTLA-4 plus PD-1 cohorts (6, 21, 23), and two CTLA-4 plus PD-L1 cohorts (14, 28). The overall pooled pCR rate was 26.8% (95% confidence interval [CI]: 19.8% - 33.7%, **Figure 2A**). In subgroup analysis, patients of ICI monotherapy and dual-ICIs therapy had a pooled pCR rate of 24% (95% CI: 15.3% - 32.8%, **Figure 2B**) and 32.1% (95%CI: 20.6% - 43.7%, **Figure 2C**), respectively. Considering the significant heterogeneity in the pooled results, we further analyzed the pooled pCR rate of PD-(L)1 monotherapy and also observed significant heterogeneity (I^2^ = 65%, P < 0.01, **Figure 2D**), which may be explained by the variations of the therapeutic strategies such as treatment cycles and dosage.

For neoadjuvant chemoimmunotherapy, 11 records (over 70% explored the GC plus ICI therapy) involving 340/389 (87.4%) RC patients were included in this analysis. The overall pooled pCR rate was 42.6% (95% CI: 34.9% - 50.2%, **Figure 2E**). The heterogeneity was significant. Therefore, we performed a subgroup analysis based on the eight studies that investigated GC plus ICI therapy. Results suggested the pooled pCR rate of 41.7% (95%CI: 35.8% - 47.5%) and no significant heterogeneity (I^2^ = 0%, P = 0.46, **Figure 2F**).

#### pPR

Nine records (ten cohorts) involving 274 RC patients investigating immunotherapy and 11 studies involving 340 RC patients investigating chemoimmunotherapy were included in this analysis.

**Figure 3:**
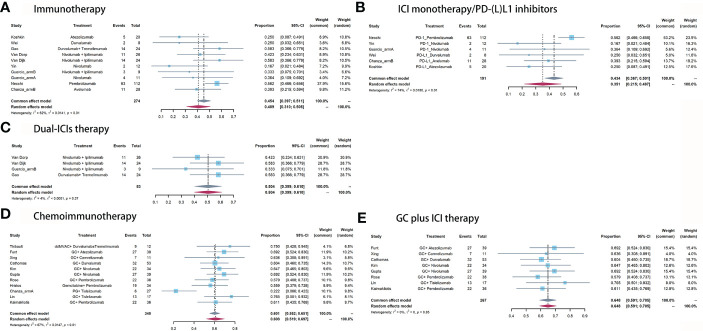
Pooled pPR of **(A)** immunotherapy, **(B)** ICI monotherapy/PD-(L)1 inhibitors, **(C)** dual-ICIs therapy, **(D)** chemoimmunotherapy, and **(E)** GC plus ICI therapy.

For immunotherapy, the overall pooled pPR rate was 40.9% (95% CI: 31%-50.8%, **Figure 3A**). In subgroup analysis, the pooled pPR rate of ICI monotherapy was 35.1% (95% CI: 21.5% - 48.7%, **Figure 3B**). Although these ICIs belong to PD-(L)1 inhibitor, significant heterogeneity was also observed (I^2^ = 74%, P < 0.01). The pooled pPR rate of dual-ICIs therapy was 50.4% (95% CI: 39.9% - 61.0%, **Figure 3C**).

For chemoimmunotherapy, the overall pooled pPR rate was 60.8% (95% CI: 51.9%-69.7%, **Figure 3D**). In subgroup of GC plus ICI therapy, the pooled pPR rate was 64.8% (95% CI: 59.1%-70.5%) and no significant heterogeneity was observed (I^2^ = 0, P = 0.85, **Figure 3E**).

### Safety outcomes

The non-responder proportion was 7.7% (11/142), 4.2% (8/189), and 12.5% (12/96) in ICI monotherapy, dual-ICIs therapy, and chemoimmunotherapy, respectively (**Table 2**). The higher rate in dual-ICIs therapy may be explained by the hyperprogression (35).

**Figure 4:**
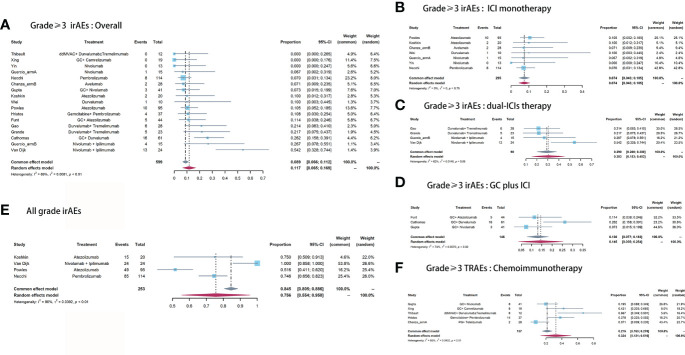
Pooled Grade≥ 3 irAEs rate of **(A)** overall, **(B)** ICI monotherapy, **(C)** dual-ICIs therapy, **(D)** GC plus ICI therapy. **(E)** Pooled all grade irAEs rate. **(F)** Pooled Grade≥ 3 TRAEs rate of chemoimmunotherapy.

Across these included studies, Grade≥3 irAEs (17 cohorts) were more widely reported than either all grade irAEs (four cohorts) or TRAEs (five cohorts). The Grade≥3 irAEs morbidity ranged from 0 (22) to 54.2% (6). Nine of the 16 studies reported the Grade≥3 irAEs in detail. The top three most common Grade≥3 irAEs were hepatitis (4.7%), pancreatitis (4.3%), and immune-mediated diarrhea and colitis (imDC, 2.3%, **Table 3**). The pooled morbidity of Grade≥3 and all grade irAEs was 11.7% (95% CI: 6.5%-16.9%, **Figure 4A**) and 75.6% (95% CI: 55.4%-95.8%, **Figure 4E**), respectively. In subgroup analysis, the pooled Grade≥3 irAEs rate for ICI monotherapy, dual-ICIs therapy, and GC plus ICI therapy was 7.4% (95% CI: 4.3%-10.5%, **Figure 4B**), 30.3% (95% CI: 15.3%-45.3%, **Figure 4C**), and 14.5% (95% CI: 3.5% - 25.4%, **Figure 4D**), respectively.

**Table 3:** AEs reported in included studies. .

		Number of reported studies	Events	Morbidity
≥ Grade 3 irAEs (Immunotherapy and chemoimmunotherapy)	Liver enzymes increase	5 (6, 20, 21, 28, 34)	14	4.7%
	Amylase/lipase increase	4 (6, 20, 21, 28)	13	4.3%
	imDC	3 (6, 20, 28)	7	2.3%
	Hematological toxicity	3 (6, 18, 28)	4	1.3%
	Skin reaction	2 (6, 20)	3	1.0%
	Electrolyte disorder	2 (20, 28)	3	1.0%
	Neuropathy	2 (6, 7)	2	0.7%
	Fatigue	2 (6, 21)	2	0.7%
	Pneumonitis	2 (21, 34)	2	0.7%
	Adenitis	1 (7)	2	0.7%
	Xerostomia/Sjögren syndrome	1 (20)	1	0.3%
	Myocarditis	1 (21)	1	0.3%
	Hyperglycemia	1 (6)	1	0.3%
≥ Grade 3 TRAEs (Chemoimmunotherapy only)	Hematological disorders		19	38.8%
	Fatigue		2	4.1%
	Anal abscess		1	2.0%
	Renal insufficiency		1	2.0%

irAEs, immune-related adverse events; TRAEs, treatment-related adverse events; imDC, immune-mediated diarrhea and colitis. ≥ Grade 3 irAEs were reported in both immunotherapy and chemoimmunotherapy studies, while ≥ Grade 3 TRAEs were only reported in chemotherapy studies.

To control the irAEs, the systemic steroid was required for two, four, and four patients in the studies by Funt et al. Necchi et al., and Gao et al, respectively (20, 25, 28). Only the ABACUS study reported one treatment-related death (16).

To assess the cumulative toxicity of chemotherapy, we further extracted the Grade≥ 3 TRAEs in patients receiving chemoimmunotherapy. The pooled morbidity was 32.4% (95% CI: 13.1% - 51.6%, **Figure 4F**). The hematological disorder was the most common Grade≥ 3 TRAEs, with morbidity of 38.8%. The detailed Grade≥ 3 irAEs and TRAEs were demonstrated in **Table 3**.

Three studies reported surgery-related complications (16, 20, 28). The postoperative all grade and Grade≥3 complication rate was 57.8% and 18.4%, respectively.

### Survival outcomes

No long-term survival data was available and no adequate time-to-event data existed for pooled analysis. **Table 4** demonstrated the survival data in detail. The two most reported survival outcomes were 1yr or 2yr OS rate and RFS rate. 1yr RFS rate varied from 68% (21) to 84.4% (7). 1yr OS rate varied from 88.8% (28) to 94% (17). Only two studies reported a 2yr OS rate and RFS rate (17, 30).

**Table 4:** Most frequently reported survival data.

Author	Study arm(s)	1yr RFS	1yr OS	2yr RFS	2yr OS
Gupta (7)	GC+ Nivolumab	85.40%			
Guercio_armA (21)	Nivolumab	77%			
Guercio_armB (21)	Nivolumab + Ipilimumab	68%			
Powles (16)	Atezolizumab	79%			
Koshkin (17)	Atezolizumab	71%	94%	64%	75%
Gao (28)	Durvalumab+ Tremelimumab	82.80%	88.80%		
Cathomas (30)	GC+ Durvalumab			83.50%	87.30%
Hristos (32)	Gemcitabine+ Pembrolizumab	74.90%	93.80%		

RFS, recurrence free survival; OS, overall survival; GC, gemcitabine/cisplatin. Any box left blank is related to information that has not been reported.

### Publication bias and influence analysis

**Figure 5:**
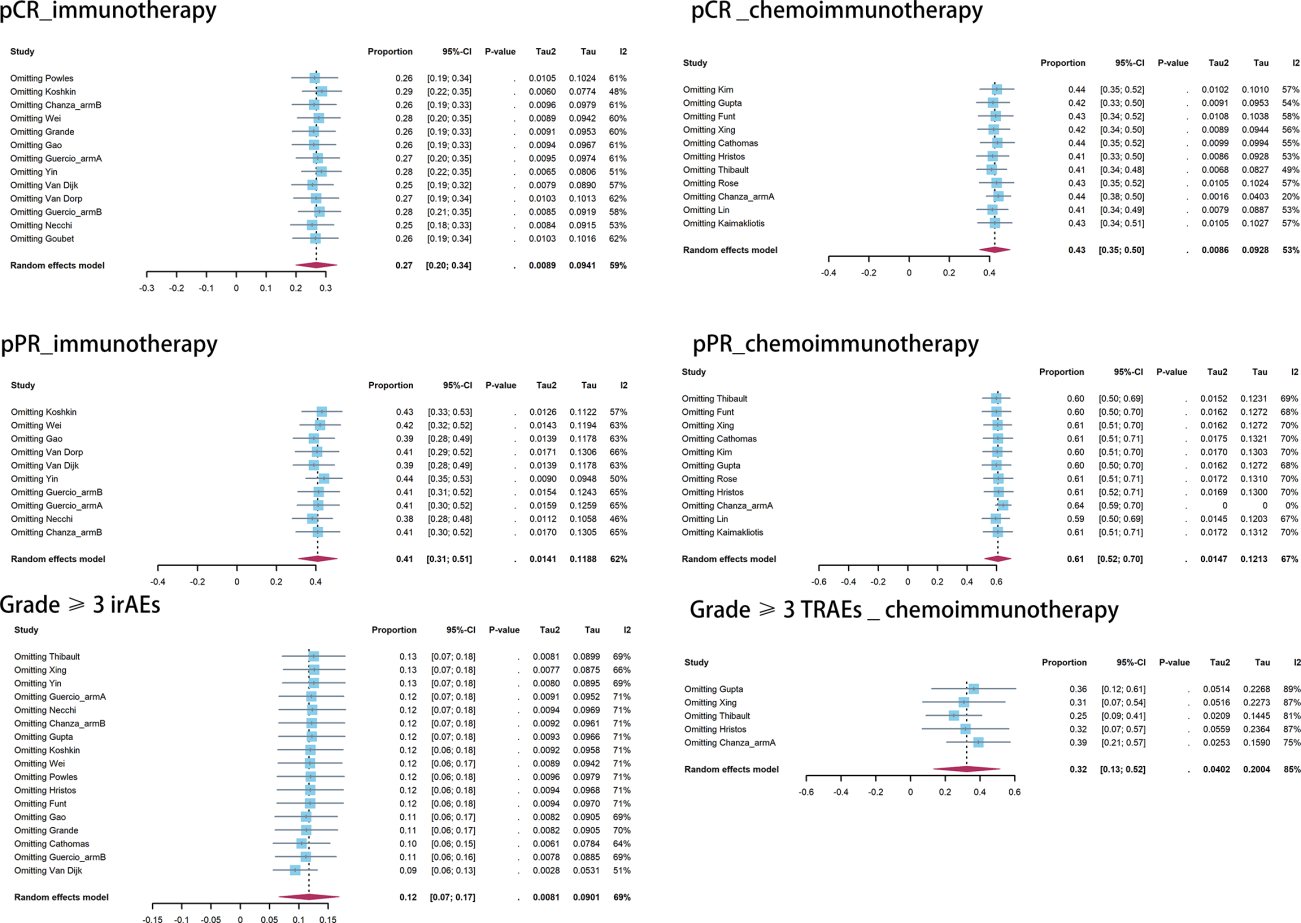
Influence analysis.

Publication bias was observed in the pooled pCR of chemoimmunotherapy and the pooled pPR of immunotherapy (**Table 5**). Influence analysis for Grade≥ 3 irAEs and Grade≥ 3 TRAEs revealed the most influential study (if omitted from the analysis, **Figure 5**).

**Table 5:** Publication bias by Egger’s regression test (only P value presented).

	pCR	pPR	irAEs
Immunotherapy	0.41	**0.05**	
ICI monotherapy	0.15	**0.03**	0.85
dual-ICIs therapy	0.80	0.41	0.47
PD-(L)1 inhibitors	0.32	**0.03**	
Chemoimmunotherapy	**0.04**	0.60	
GC plus ICI therapy	0.15	0.91	0.18

pCR, pathological complete response; pPR, pathological partial response; irAEs, immune-related adverse effects; ICI, immune checkpoint inhibitor; GC, gemcitabine/cisplatin. Any box left blank is related to information that has not been reported. Values in bold indicated significant publication bias of corresponding terms.

## Discussion

Given the remarkable evidence revealed by the IMvigor 210 (36) and CheckMate 275 studies (37), there has been growing interest in investigating ICIs in the neoadjuvant treatment for patients with MIBC. To date, three main types of immunological therapies have been widely evaluated in the neoadjuvant setting: ICI monotherapy, dual-ICIs therapy, and chemoimmunotherapy (dominated by GC plus ICI therapy).

### Role of ICI immunotherapy

For cisplatin-ineligible patients, ICIs treatment has shaped the therapeutic paradigm. Compared to ICI monotherapy, dual-ICIs therapy (combined inhibition of PD-1/PD-L1 and CTLA-4) can enhance the therapeutic efficacy. In our study, the pooled pCR of dual-ICIs therapy was slightly higher than that of ICI monotherapy (32.1% vs 24%). The superiority of dual-ICIs therapy to ICI monotherapy was more evident in terms of the pooled pPR (50.4% vs 35.1%). In one recent meta-analysis involving 16 studies of 988 participants (38), Jiang et al. comprehensively integrated the evidence of neoadjuvant immunotherapy or chemoimmunotherapy in non-small cell lung cancer (NSCLC) and reported that the pooled pCR (ypT0N0M0) for ICI monotherapy and dual-ICIs therapy was 9.9% (95% CI: 5.7% - 15.3%) and 28.6% (13.8% - 50.7%), respectively.

Blocking PD-(L)1 and CTLA-4 simultaneously can destroy the immune suppression induced by tumor cells and enhance tumor rejection *via* effective T cells infiltration, activation, survival, and proliferation (39). Several studies have widely investigated the efficacy of nivolumab and ipilimumab in the treatment of melanoma and lung cancer in comparison to monotherapy. These findings suggested a significantly higher response rate and more favorable prognosis of dual-ICIs therapy, compared to ICI monotherapy (40, 41). Based on the remarkable findings uncovered by studies on melanoma and lung cancer, there have been a large number of studies exploring combined PD-(L)1 and CTLA-4 inhibition in other tumor types including MIBC, and PD-(L)1 inhibitors in combination with other immune checkpoints inhibitors. For example, Yin et al. in a prospective non-randomized study involving 43 MIBC patients, explored the efficacy between nivolumab monotherapy and combination immunotherapy of nivolumab and KIR inhibitor lirilumab. They found that the pCR of combination immunotherapy was 18%, higher than that of monotherapy (8%) (22). Small sample size may be responsible for the underestimated results. Collectively, patients receiving neoadjuvant dual-ICIs therapy can obtain more clinical benefits.

On the other hand, higher rates of pCR and pPR were associated with more frequent Grade≥ 3 irAEs. Patients who received ICI monotherapy reported a Grade≥ 3 irAEs morbidity of 7.4%, while those who received dual-ICIs therapy had a rate of 30.3%. One meta-analysis involving 2410 patients with advanced solid cancers, also reported a significantly higher irAEs rate induced by dual-ICIs therapy, compared to ICI monotherapy (42). Therefore, oncologists have to weigh the advantages and disadvantages of dual-ICIs therapy for cisplatin-ineligible patients. Future well-designed RCTs are needed to draw more robust conclusions in this concern.

### Role of chemoimmunotherapy

For cancers in the advanced or metastatic setting, chemoimmunotherapy has demonstrated a more favorable OS and PFS, compared to chemotherapy alone. The IMvigor130 study explored survival differences between atezolizumab plus chemotherapy (n=451) and chemotherapy alone (n=400). Results revealed an 18% PFS and a 17% OS benefit for those who received chemoimmunotherapy combinations compared with those who received chemotherapy alone (43). More significant findings have been reported in the JAVELIN Bladder 100 study (44). In the phase III RCT trial, 700 patients were allocated to maintenance avelumab plus best supportive care and best supportive care alone in a 1:1 ratio. Patients receiving combinatory treatment achieved a 31% OS and 38% PFS benefit. Intriguing results promoted investigators to evaluate the efficacy of chemoimmunotherapy in the neoadjuvant setting.

In our study, the pooled pCR and pPR for chemoimmunotherapy was 42.6% and 60.8%, respectively, comparable to that of NAC (45). Considering the establishment of effective immune memory, immunotherapy was featured for the long-lasting anti-tumor efficacy, compared to chemotherapy. Compared to patients receiving traditional NAC, whether patients receiving chemoimmunotherapy had a more favorable prognosis or not remains unclear, since these enrolled patients are under active long-term follow-up in terms of survival. Promisingly, several studies have uncovered that achieving pCR in patients receiving NAC was associated with a favorable prognosis (46, 47), suggesting the expectation of clinical benefits of neoadjuvant chemoimmunotherapy in survival outcomes.

In comparison to ICI immunotherapy, chemoimmunotherapy had a comparable pCR to dual-ICIs therapy and a higher pCR to ICI monotherapy, consistent with the findings uncovered by Jiang et al. in their study of NSCLC. Compared to inhibition of a single immune checkpoint, the addition of chemotherapy can destroy the tumor cells and release large amounts of tumor antigen at the time of high tumor burden (preoperative setting). Therefore, the efficacy of immunotherapy can be promoted (48). When interpreting these results, we must keep in mind that the participants’ selection bias may skew the results. Most participants receiving ICI immunotherapy are cisplatin-ineligible and have worse physic conditions compared to participants receiving chemoimmunotherapy, as impaired physic conditions (evaluated by ECOG PS score) correlated with limited response to immunotherapy and unfavorable prognosis (49).

In terms of safety, the Grade≥ 3 irAEs morbidity was much lower than that of dual-ICIs therapy. However, the Grade≥ 3 TRAEs rate of chemoimmunotherapy was 32.4% (ranging from 7.1% to 66.7%), comparable to that of NAC in MIBC (50).

### Neoadjuvant ICI combined with targeted therapy: the rational and current progress

Targeted therapy is designed to interfere with critical biological pathways (such as RTK/RAS/MAP-Kinase pathway and I3K/Akt signaling) and block dysregulated proteins (such as EGFR and BRAF) which play essential roles in tumor formation, growth, and invasion (51). Although the impressive anti-tumor effects of various targeted agents in selected patients have been well-documented, the rapid emergence of resistance has become a major concern due to, for example, the restoration of the pathways or biological functions by activation of upstream or downstream effectors (52). Therefore, a novel treatment strategy is important to prolong the anti-tumor effect and amplify the prognostic benefits. Preliminary findings have addressed the potent anti-tumor immunity by ICIs in intractable cancers including metastatic melanoma and metastatic urothelial carcinoma (53, 54). More importantly, ICI therapy demonstrated long-term benefits due to restored host anti-tumor response and sustained immune memory. The weaknesses of targeted therapy and strengths of ICIs have inspired the idea of combinatorial therapy that can benefit patients by its synergistic effect:

(1) The immunosuppressive tumor microenvironment (TME) is a hallmark of most malignancies and hinders anti-tumor immunity. Targeted therapy can induce rapid destruction of this feature and enhance the cytotoxicity of immunotherapy (55);
(2) The tumor antigen released by targeted therapy can act as tumor vaccinations to consolidate the host immune response and anti-tumor effects (56);
(3) By inhibiting the regulatory T cells (Tregs) and myeloid-derived suppressor cells (MDSCs) and improving the effects of antigen-presenting DC, targeted therapy can restore the activity of effector T cells (57).

Currently, there was only one study by Rodriguez-Moreno and colleagues (58) evaluating the combinatorial therapy (durvalumab in 1500 mg q4wks and Olaparib in 500 mg bid orally, followed by RC within 8-10 wks) for MIBC in the neoadjuvant setting. After 28 patients with cT2-T4a MIBC enrolled, 12 of them underwent RC and 44.5% achieved a pCR rate of 44.5%. In terms of safety, one surgery-related death was reported. Although over 90% of patients presented with any grade of AEs, only 8.3% suffered from Grade 3-4 AEs. Besides, no Grade≥3 Olaparib-related AEs were observed. Limited by the single-arm design and sample size, robust conclusions were hard to be addressed.

### Strengths and limitations

Although there have been several published reviews/meta-analyses addressing this concern, our study has some strengths. First, considering the rapid development of immunotherapy, we performed the literature research up to June 2022 after the 2022 ASCO Annual Meeting to search for the latest eligible records. Second, we performed strict literature identification and only included the updated results of one trail to avoid duplicate records. Third, given the discrepancies in included studies, we pooled the oncological outcomes by PP analysis, which can guide the counsel of patients. Fourth, we performed subgroup analysis according to the types of treatment regimens, which can represent the current progress of each approach to some extent.

There were some limitations in our study. First, although subgroup analysis was performed, significant heterogeneity still presented in the majority of outcomes of our study, which may be caused by the variations in dosage and cycles of regimens and the included population bias (varied eligibility to cisplatin and physic performance). Second, most included trials were single-arm designed, with small sample size, which prevented drawing robust recommendations. Third, the lack of long-term survival data made it hard to know the lasting benefits of immunotherapy in the neoadjuvant setting.

## Conclusions

The three main immunotherapeutic approaches, including ICI monotherapy, dual-ICIs therapy, and chemoimmunotherapy (dominated by GC plus ICI therapy), were effective and safe in the treatment of MIBC in the neoadjuvant setting. Compared to ICI monotherapy, dual-ICIs therapy and chemoimmunotherapy had higher pCR and pPR. On the other hand, the morbidity of Grade≥ 3 irAEs or Grade≥ 3 TRAEs of dual-ICIs therapy and chemoimmunotherapy was higher than that of ICI monotherapy. Current single-arm designed studies with missing long-term prognostic data, however, prevented us from drawing reliable recommendations. Oncologists must weigh the pros and cons before deciding on one specific therapeutic strategy.

## Data availability statement

The original contributions presented in the study are included in the article/supplementary material. Further inquiries can be directed to the corresponding author.

## Author contributions

Study design and registration: HLC. and XQX. Search strategy design: HLC. and YJL. literature search: HLC. and ZHJ. Study selection: HLC. and WJY. Data collection and extraction: HLC. and WJY. Data analysis and writing: HLC. Arrangement and supervision: ZGJ. All authors contributed to the article and approved the submitted version

## Conflict of interest

The authors declare that the research was conducted in the absence of any commercial or financial relationships that could be construed as a potential conflict of interest.

## Publisher’s note

All claims expressed in this article are solely those of the authors and do not necessarily represent those of their affiliated organizations, or those of the publisher, the editors and the reviewers. Any product that may be evaluated in this article, or claim that may be made by its manufacturer, is not guaranteed or endorsed by the publisher.
